# Designing CXCL8-based decoy proteins with strong anti-inflammatory activity *in vivo*

**DOI:** 10.1042/BSR20130069

**Published:** 2013-09-17

**Authors:** Angelika Falsone, Veronica Wabitsch, Elena Geretti, Heide Potzinger, Tanja Gerlza, James Robinson, Tiziana Adage, Mauro M. Teixeira, Andreas J. Kungl

**Affiliations:** *ProtAffin Biotechnologie AG, Reininghausstrasse 13a, A-8020 Graz, Austria; †Institute of Pharmaceutical Sciences, University of Graz, Universitätsplatz 1, A-8010 Graz, Austria; ‡Universidade Federal de Minas Gerais, Departamento de Bioquimica e Imunologia, Av. Antonio Carlos, 6627–Pampulha, 31270-901 Belo Horizonte, Brazil

**Keywords:** anti-inflammatory, chemokine decoys, competition, GAG affinity, heparan sulphate, AIA, antigen induced arthritis, CCL, CC chemokine ligand, CS, chondroitin sulphate, CXCL, CXC chemokine ligand, CXCR, CXC chemokine receptor, GAG, glycosaminoglycan, GPCR, G-protein-coupled receptor, HBSS, Hanks balanced salt solution, HS, heparan sulphate, IFT, isothermal fluorescence titration, IL, interleukin, *K*d, dissociation constant, KC, keratinocyte chemoattractant, LMWH, low molecular weight heparin, mBSA, methylated BSA, MIP, macrophage inflammatory protein, MPO, myeloperoxidase, RA, rheumatoid arthritis, SP, sulphopropyl, SPR, surface plasmon resonance, TFA, trifluoroacetic acid, TMB, tetramethylbenzidine, UFMG, Universidade Federal de Minas Gerais, WT, wild-type

## Abstract

IL (interleukin)-8 [CXCL8 (CXC chemokine ligand 8)] exerts its role in inflammation by triggering neutrophils via its specific GPCRs (G-protein-coupled receptors), CXCR1 (CXC chemokine receptor 1) and CXCR2, for which additional binding to endothelial HS-GAGs (heparan sulphate-glycosaminoglycans) is required. We present here a novel approach for blocking the CXCL8-related inflammatory cascade by generating dominant-negative CXCL8 mutants with improved GAG-binding affinity and knocked-out CXCR1/CXCR2 activity. These non-signalling CXCL8 decoy proteins are able to displace WT (wild-type) CXCL8 and to prevent CXCR1/CXCR2 signalling thereby interfering with the inflammatory response. We have designed 14 CXCL8 mutants that we subdivided into three classes according to number and site of mutations. The decoys were characterized by IFTs (isothermal fluorescence titrations) and SPR (surface plasmon resonance) to determine GAG affinity. Protein stability and structural changes were evaluated by far-UV CD spectroscopy and knocked-out GPCR response was shown by Boyden chamber and Ca^2+^ release assays. From these experiments, CXCL8(Δ6F17KF21KE70KN71K) emerged with the most promising *in vitro* characteristics. This mutant was therefore further investigated in a murine model of mBSA (methylated BSA)-induced arthritis in mice where it showed strong anti-inflammatory activity. Based on these results, we propose that dominant-negative CXCL8 decoy proteins are a promising class of novel biopharmaceuticals with high therapeutic potential in inflammatory diseases.

## INTRODUCTION

Chemokines are important key mediators in inflammation. They are responsible for leucocyte attraction, activation and diapedesis across the endothelium from the blood stream into the affected tissue. Once bound to the HS–GAG (heparan sulphate–glycosaminoglycan) chains of endothelial cell surface proteoglycans of blood vessels [1] they are known to undergo structural changes [2]. Binding, immobilization, structural reorganization and oligomerization *via* GAG co-receptors were shown to be major prerequisites for leucocyte receptor binding and activation, which finally leads to leucocyte extravasation into the inflamed tissue whereby host defence mechanisms are induced to resolve injury [3]. Inappropriate regulation, however, can lead to pathological situations, e.g. various chronic inflammatory diseases or autoimmune diseases, which are also characterized by changes in GAG patterns [4,5].

IL (interleukin)-8 [CXCL8 (CXC chemokine ligand 8)] is an 8 kDa CXC-chemokine that attracts neutrophils to sites of inflammation when immobilized on endothelial GAG chains in the vasculature [1]. The subsequent binding of the chemokine to the neutrophil GPCRs (G-protein coupled receptors), CXCR1 (CXC chemokine receptor 1) and CXCR2, fully activates the already slowed down (selectin-mediated ‘rolling’) neutrophil and leads to firm adhesion and subsequent transmigration through the blood vessel endothelium into the tissue [6]. The three-dimensional solution structure of CXCL8 shows a dimer with two symmetry-related, antiparallel α-helices, which lie on top of six-stranded antiparallel β-sheets derived from two three-stranded Greek keys, one from each monomer unit. Dimerization is not a prerequisite for full inflammatory activity as CXCL8 monomeric variants are known to exhibit comparable inflammatory activity *in vitro* and *in vivo* [7]. Despite its small size, CXCL8 exhibits discrete but connected structural domains by which the chemokine interacts with its two biological receptors: with the traditional GPCRs CXCR1 and CXCR2 on the one hand [8] and with GAG co-receptors on the other hand [9].

GAGs such as HS, heparin or CS (chondroitin sulphate) are long, linear, heterogeneous and highly negatively charged (sulphated) polysaccharide chains composed of repeating disaccharide units that are commonly covalently connected (O-linked) to core proteins, thus forming the so-called proteoglycans, which are located at the cell surface of virtually all eukaryotic cells constituting the glycocalyx. The interaction of proteins with GAGs is driven by the strong electrostatic forces between the sulphate groups on the carbohydrate and the amine groups of lysine, arginine and histidine residues on the protein. Specificity of the GAG–protein interaction is introduced *via* low-affinity hydrogen bonding and van der Waal's forces between the two molecules [10]. Taken together with the high flexibility of typical GAG chains, which results in high binding entropies, the affinity between proteins and GAGs is commonly observed in the low micromolar range. Among the multitude of GAG-binding proteins, the chemokines are one of the best characterized protein class with respect to these complex ligands [11,12].

The residues of CXCL8 responsible for GAG binding have been identified by site-directed mutagenesis and NMR experiments to include Arg^60^, Lys^64^, Lys^67^ and Arg^68^ that are contained in the C-terminal α-helix as well as His^18^ and Lys^20^ contained within the proximal loop [9]. These results were further confirmed in our own recent work by molecular modelling studies and IFT (isothermal fluorescence titration) experiments [13,2]. The affinity of CXCL8 for heparin and HS was found to be dependent on the oligomerization state of the chemokine being significantly higher for the monomer compared with the dimeric form of the chemokine [2]. For CXCR1 and CXCR2 binding and signalling, the N-terminal ELR motif is essential. In addition, a hydrophobic pocket in the proximal N-loop (Ile^10^, Tyr^13^, Phe^17^, Phe^21^, Ile^22^) was found to contribute to the GPCR interaction [14]. Apparently, the proximal loop provides a topological and functional interface for CXCL8 binding to its GPCR(s) as well as to its GAG co-receptor.

Here we report the design, expression and characterization of a set of CXCL8 mutants comprising three classes of different engineering sites within the chemokine and their combination. We have recently published the engineering of increased GAG-binding affinity into two chemokines, namely CCL2 (CC chemokine ligand 2) and CCL5 [17,18]. The underlying study, however, represents the first GAG-directed engineering approach applied to a CXC chemokine, namely the prototypic member of this class CXCL8. CXC chemokines, as the smaller chemokine sub-family, seem to be structurally much more homogeneous than CC chemokines and therefore ‘easier’ to modify. Our study on CXCL8 should pave the way for structure-based affinity engineering of the entire chemokine class. Preceding *in silico* analysis based on IL8/GAG docking studies using the YASARA NOVA force field [13], was performed to identify the amino acids to be mutated and to build up a set of mutants that were expressed in *Escherichia coli* and subsequently biophysically characterized. The docking studies took explicitly into account the mobility of side-chain residues in order to allow conformational adjustment following ligand binding. Our evaluation criteria for the various CXCL8 mutants were: (i) IFT measurements and SPR (surface plasmon resonance) measurements to investigate enhanced GAG binding; (ii) CD spectroscopy to prove a conserved chemokine fold as well as structural changes upon GAG binding; (iii) guanidine unfolding to demonstrate structural homogeneity and stability; and (iv) Boyden chamber plus Ca^2+^ release assays to check for chemotactic knock-out of the protein variants. Having applied these evaluation criteria, the CXCL8(Δ6F17KF21KE70KN71K) decoy protein was identified as the most promising CXCL8 mutant that was therefore further investigated in an acute (mBSA (methylated BSA)-induced) murine arthritis model.

## MATERIALS AND METHODS

All chemicals, unless stated otherwise, were obtained from Sigma-Aldrich. The Sigma HS (H7640) used for all the experiments was isolated from bovine kidney and has an average molecular mass of 12 000 Da. The LMWH (low molecular weight heparin; H4784) from pig mucosa with an average molecular mass of 5 000 Da was also purchased from Sigma.

### Cloning, expression and purification of wtCXCL8 and CXCL8 mutants

Using a synthetic, *E. coli* codon-optimized *CXCL8* gene (Table 1), we performed PCR-aided site-directed mutagenesis for the introduction of the desired amino acid replacements. PCR was performed on a Mastercycler Gradient (Eppendorf) using primers from Invitrogen and the enzyme TITANIUM™ *Taq* DNA polymerase (Advantage® 2 Polymerase Mix, CLONTECH).

**Table 1:** The three classes of designed CXCL8 mutants including a gradation of the type of mutation +, strong mutation; ~, light mutation.

CXCL8 mutant		Type/location of mutation
CXCL8(F21R)	1.class of mutants	**~**/ loop
CXCL8(F21K)		**+**/ loop
CXCL8(F17K)		**+**/ loop
CXCL8(F17RF21R)		**~~**/ loop
CXCL8(F17KF21R)		**+~**/ loop
CXCL8(F17RF21K)		**~+**/ loop
CXCL8(F17KF21K)		**++/** loop
CXCL8(Δ6E70R)	2.class of mutants	**~/** -ELR, α-helix
CXCL8(Δ6E70KN71K)		**++/** -ELR, α-helix
CXCL8(Δ6F17RF21RE70K)	3.class of mutants	**~~+/** -ELR, loop, α-helix
CXCL8(Δ6F17RF21RN71K)		**~~+/** -ELR, loop, α-helix
CXCL8(Δ6F17RF21RE70KN71R)		**~~+~/** -ELR, loop, α-helix
CXCL8(Δ6F17RF21RE70RN71K)		**~~~+/** -ELR, loop, α-helix
CXCL8(Δ6F17KF21KE70KN71K)		**++++/** -ELR, loop, α-helix

Directional cloning in pET101/D-TOPO (Invitrogen) and subsequent transformation into calcium competent BL21 Star (DE3) *E. coli* cells was carried out using the pET Directional TOPO Expression Kit (Invitrogen). Correct sequences were verified by double-stranded DNA sequencing with an ABI PRISM 310 Sequencer (Applied Biosystems) prior to expression and purification. The mutants thus obtained are summarized in Table 1 emphasizing once more the location of the respective mutations.

For mutant as well as wtCXCL8 expression, an overnight culture of BL21 Star (DE3) *E. coli*, produced from a glycerol stock, was diluted 1:125 with LB (Luria–Bertani) medium containing 100 μg/ml ampicillin (AppliChem) and agitated vigorously until an OD of 0.8 was reached. Growth conditions were: 37°C and 200 rev./min. Expression was induced by adding 1 mM IPTG (isopropyl β-d-thiogalactopyranoside; AppliChem) and the incubation was extended for another 4 h. Cells were centrifuged at 6000 ***g*** for 20 min, resuspended in 20 mM Tris/HCl, 50 mM NaCl; pH 8 (2 ml/g pellet) and lysed on ice by sonication. The lysates were centrifuged at 10000 ***g*** for 20 min at 4°C. One gramme of pellet (containing inclusion bodies) was resuspended in 10 ml of 6 M GdnHCl (guanidinium chloride) (50 mM MES, pH 6.5) for 4 h at 4°C. After centrifugation at 10000 ***g*** for 20 min the supernatant underwent extensive dialysis against 0.5% (v/v) CH_3_COOH overnight at 4°C. The centrifuged dialysate was subjected to cationic exchange chromatography using an SP (sulphopropyl) sepharose column, a strong cation exchanger (GE Healthcare) and a linear gradient from 50 mM MES (pH 6.5) to 50 mM MES, 1 M NaCl (pH 6.5). This was followed by RP-HPLC (reversed-phase HPLC) on a C18 column, which was performed applying a non-linear gradient: from 10 to 46% acetonitrile (Fisher Scientific) containing 0.1% (v/v) TFA (trifluoroacetic acid) in 5 min, from 46 to 64% (v/v) acetonitrile containing 0.1% TFA in 20 min and from 64 to 100% acetonitrile containing 0.1% TFA in 5 min. Refolding was accomplished on an SP sepharose column as described above, but in addition under endotoxin-free conditions. Therefore glassware was heat-sterilized at 180°C at least for 3 h and the whole setup was flushed with 1 M NaOH for 1 h before usage. Fractions were concentrated using Centriplus centrifugal filter devices (Millipore). Contamination with LPSs (lipopolysaccharides) was determined by the LAL Reagent Kit (Cambrex) and was found to be less than 0.18 EU (endotoxin units)/ml.

### Fluorescence measurements and data analysis

The fluorescence measurements were performed on a PerkinElmer LS50B fluorometer (Beaconsfield).

### IFT experiments

The emission of 0.7 μM pre-equilibrated protein solutions in PBS upon excitation at 282 nm was recorded over the range of 310–400 nm. The spectra were recorded at a speed of 200 nm/min and a 290 nm cut-off filter was inserted into the emission path to avoid stray light. The temperature was maintained at 20°C by coupling to an external water bath. An equilibration period of 1 min followed each addition of heparin/HS. Concentrated heparin/HS stock solutions were used for the titration experiments to ensure a protein dilution of less than 3%. Two independent measurements were performed. After background subtraction and integration the normalized mean changes in fluorescence intensity (−Δ*F*/*F*_0_) were plotted against the ligand concentration. The resulting binding isotherms were analysed by non-linear regression using the program Origin 5.0 (Microcal Inc.) to the following equation describing a bimolecular association reaction:

(1) $$\begin{array}{ccc} F\; & = & \;F_{i}\;+\;F_{\max}\;\cdot \;\frac{K_{d}\;+\;\left[P\right]\;+\;\left[L\right]\;-\sqrt{{\left(K_{d}\;+\;\left[P\right]\;+\;\left[L\right]\right)}^{2}\;-\;4\left[P\right]\left[L\right]}}{2[P]} \end{array}$$

where *F*_i_ is the initial and *F*_max_ is the maximum fluorescence value, *K*_d_ (dissociation constant); [*P*] and [*L*] are the total concentrations of the protein under investigation and the respective ligand. The fitted parameters were *F*_max_ and *K*_d_. This equation is based on the general solution for a bimolecular association reaction [2].

#### Equilibrium denaturation experiments

0.7 μM protein solutions at different guanidine (MP Biomedicals) concentrations in the range of 0–6 M guanidine were prepared and equilibrated for 30 min at room temperature (°C) Fluorescence scans were recorded as described above and the wavelength-shift of the protein upon increasing guanidine concentration was analysed using the Boltzmann equation:

(2) $${y=A}_{2}+\left(A_{1}-A_{2}\right)/\left\{1+\exp\left[({x-x}_{0})/\mathrm{dx}\right]\right\}$$

where *A*_1_ is the initial and *A*_2_ the final wavelength. The fitted parameters are *dx* and *x*_0._

### SPR

Binding was investigated on a BIAcore 3000 instrument (BIAcore AB). The immobilization of biotinylated HS onto a streptavidin coated CM4 sensor chip was performed according to an established protocol, described recently [15]. The binding interactions were recorded at 25°C in PBS pH 7.4 containing 0.01% (v/v) P20 surfactant (BIAcore AB). 4 min injections of different protein concentrations at a flow rate of 30 μl/min were followed by 10 min dissociation periods in buffer and a pulse of 1 M sodium chloride for complete regeneration. The maximum response signals of protein binding to the GAG surface, corresponding to the plateaus of the respective sensorgrams, were used for Scatchard plot analysis and the calculation of equilibrium *K*_d_ values.

### Biotinylation of proteins

Buffer exchange was performed to 0.1 M MES pH 5 using Amicon Ultracel 3K 4 ml (Millipore) to provide optimum reaction conditions for biotinylation. wtCXCL8 was incubated with 20 M excess of EZ Link Penthylamine Biotin (Thermo Scientific) and 10 M excess EDC (Pierce) for 2 h at room temperature, low agitation. Desalting was performed using ZEBA desalting columns (Pierce) according to manufacturer's protocol. Biotinylation grade was determined using a Biotin Quantification kit (Pierce) and protein concentration was determined using photometric analysis.

### Competition assay

2.5 μg HS were coated on specially prepared Iduron plates (Iduron) that adsorb GAGs without modifications, whilst retaining their protein-binding characteristics, over night at room temperature. After washing the plate using an automatic platewasher (Tecan) with PBS (137 mM NaCl, 2.7 mM KCl, 8.1 mM Na_2_HPO_4_, 1.45 mM KH_2_PO_4_, pH 7.2), it was incubated with 250 nM biotinylated IL-8 diluted in PBS at room temperature. After 1 h the plate was washed to remove unbound biotinylated CXCL8 and incubated with different competitor concentrations starting from 50 to 3 nM diluted in PBS for 30 min at room temperature. Each concentration was measured in triplicate. To detect the remaining biotinylated CXCL8 we used an ELISA-like setup. After another washing step we incubated the plates with streptavidin HRP (horseradish peroxidase) (Pierce) diluted in 0.2% dry milk that binds to the biotinylated CXCL8 in the plate that was not competed off by the competitor. After another hour of incubation at room temperature and removal of unbound Streptavidin by washing, we analysed the plate by adding the substrate TMB (tetramethylbenzidine), resulting in a blue colour change. After stopping the reaction with sulphuric acid the absorbance at 450 nm was measured using Beckman Coulter DTX 800 Multimode Detector (Beckman), with correction at 620 nm. The reference (OD620) values were subtracted from the sample values (OD450) and the mean±S.D. of the replicates was calculated. Data analysis was performed using specialized statistical software Origin 8.0 (Microcal Inc.). Data were fitted using an Origin four-parameter logistic function to calculate IC50 values.

### Preparation of human neutrophils

Human whole blood was obtained from healthy volunteers through venipuncture using heparinized tubes (Vacuette, GBO). Neutrophils were isolated by dextran sedimentation (1%) for 45 min at room temperature. The PMN (polymorphonuclear cell)-rich supernatant was aspirated and washed twice with HBSS (Hanks balanced salt solution)(−) (PAA) at 600 ***g*** for 10 min. Finally, the cells were diluted with HBSS(+) at 2×10^6^/ml, taking into account that only 60% of the counted cells were neutrophils.

### Neutrophil chemotaxis

A 48-well micro chemotaxis Boyden chamber (Neuroprobes) with a 5 μm pore size PVP-free polycarbonate membrane (Neuroprobes) was used to examine chemotaxis of neutrophils in response to wtCXCL8 and mutants. Proteins were diluted at concentrations of 10, 1 and 0.1 μg/ml and put in triplicates in the lower compartment of the chamber. Approximately 50 μl of the freshly prepared neutrophils (1.0×10^5^ cells) were seeded in the upper chamber and incubated for 30 min at 37°C in 5% (v/v) CO_2_ humidified incubator. After incubation the filter was removed from the chamber, non-migrated cells were washed away and cells attached to the lower side were fixed with methanol and stained with Hemacolor solutions (Merck). Cells were then counted at ×400 magnification in four randomly selected microscopic fields per well and the chemotactic indices were calculated taking into account the background migration. Finally, the mean of three independent experiments was plotted against the chemokine concentration.

### Intracellular Ca^2+^ mobilization of neutrophils

Freshly prepared human neutrophils were incubated with 3 μM Fura-2AM in HBSS(+) supplemented with 0.5% (w/v) BSA at 37°C for half an hour. Cells were centrifuged for 3 min at 500 ***g*** and the supernatant was discarded. Excessive dye was removed by washing twice with PBS (137 mM NaCl, 2.7 mM KCl, 8.1 mM Na_2_HPO_4_, 1.5 mM KH_2_PO_4_, pH 7.3). Immediately before the fluorescent measurements, the cells were resuspended in HBSS(+) to a final concentration of 1.0×10^6^ per ml and were left for another 10 min of equilibration in the fluorimeter (PerkinElmer LS50B), which was coupled to an external water bath for ensuring 37°C throughout the measurements. The excitation wavelength was set to 340 nm and the increase of the fluorescence emission at 495 nm upon 100 nM protein induced intracellular Ca^2+^ release was recorded.

### Animal experiments

Animal care and handling procedures were performed in accordance with the guidelines of the international association for the study of pain and all the experiments received prior approval from the UFMG (Universidade Federal de Minas Gerais, Brazil) ethics committee.

### Antigen (mBSA)-induced arthritis model

The 8—10-week-old male C57Bl/6J mice obtained from Centro de Bioterismo (CEBIO) of UFMG) and maintained in the animal facilities of Laboratório de Imunofarmacologia, Department of Biochemistry and Immunology (UFMG), with filtered water, food *ad libitum* and in a controlled environment (temperature and humidity) were used in the present studies. The animals were immunized intradermally at the base of the tail with 500 μg of mBSA in 100 μl of an emulsion of saline and equal volume of Freund's complete adjuvant (CFA; Sigma) on day 0. After 14 days, antigen challenge was performed by injection of 10 μg mBSA in sterile saline (10 μl) into the left knee joint. CXCL8(Δ6F17KF21KE70KN71K) was administered subcutaneously at the doses of 0.1 and 1 μg/mouse (*n*=5/group) 30 min before the challenge with the antigen. In this experiment, hypernociception was evaluated immediately before animal sacrifice at 24 h post-challenge as previously described [16]. Mice were placed in a cage with a wire-grid floor for 15–30 min, before testing for environmental adaptation. Stimulations were performed using, an electronic pressure meter that consisted of a hand-held force transducer fitted with a polypropylene tip (4.15 mm^2^, Insight Instruments). An increasing perpendicular force was applied to the central area of the plantar surface of the hind paw to induce dorsal flexion and withdrawal. The electronic pressure meter automatically recorded the intensity of the force in grams that was applied at time of paw withdrawal. Paw withdrawal was tested before and after injection of saline or antigen, hypernociception was expressed as changes from basal.

As terminal procedure, the knee cavity was washed with PBS (2×5 μl), the total number of leucocytes in the knee lavage was determined by counting the cells in a Neubauer chamber after staining with Turk's solution. Differential count was performed on cytospin preparations (Shandon III; Thermo Shandon) by evaluating the percentage of each leucocyte population on slide stained with May–Grünwald–Giemsa stain. At time on knee lavage, the peri-articular tissue was removed, snap frozen and then homogenized for evaluation of tissue chemokines (ELISA kit, Duo-Set kits; R&D Systems) and for MPO (myeloperoxidase) activity assessment using and enzymatic assay [16]. MPO activity was used as indirect measurement of neutrophil infiltration into the tissue.

Finally, for the intravital microscopy assessment in the mBSA model, animals were treated with CXCL8(Δ6F17KF21KE70KN71K) subcutaneously at the dose of 0.1 μg/mouse (*n*=4–6) 45 min prior to the measurement that was performed 24 h post-antigen challenge. The intravital microscopy was performed in the synovial microcirculation of the mouse knee, as described previously [16]. Briefly, the hind limb of deeply anaesthetized animals was placed on a stage, the patellar tendon partly cut and the intra-articular synovial tissue visualized for the determination of leucocyte rolling and adhesion. To measure the leucocyte–endothelial cell interactions, the fluorescent marker R6G (rhodamine 6G; Sigma) was injected intravenously immediately before the measurements. Rhodamine epi-illumination was achieved with a 150 W variable HBO mercury lamp in conjunction with a Zeiss filter. The microscopic images collected under a 20-fold objective were captured with a video camera and recorded. Data analysis was performed off-line.

Rolling leucocytes were defined as those cells moving slower than the cells moving at a regular flux in a given vessel. The flux of rolling cells was measured as the number of rolling cells passing by a given point in the venule per minute, with results expressed as cells/min. A leucocyte was considered adherent if it remained stationary for at least 30 s. Total leucocyte adhesion was quantified as the number of adherent cells within a 100-μm length of venule with results expressed as cells/mm^2^.

In all the experiment, vehicle control animals were treated with PBS as the same time of administration as CXCL8(Δ6F17KF21-KE70KN71K).

Results are expressed as the mean±S.E.M. Differences between groups are evaluated by analysis of variance followed, in case of significance by Newman–Keuls for multiple comparison or Student's *t* test as *post hoc*. For both analyses a *P*<0.05 was set for significance.

## RESULTS AND DISCUSSION

**Figure 1:**
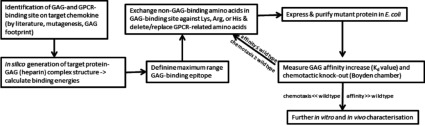
Flow chart of the structure-based design process to generate dominant-negative chemokine mutants

**Figure 2:**
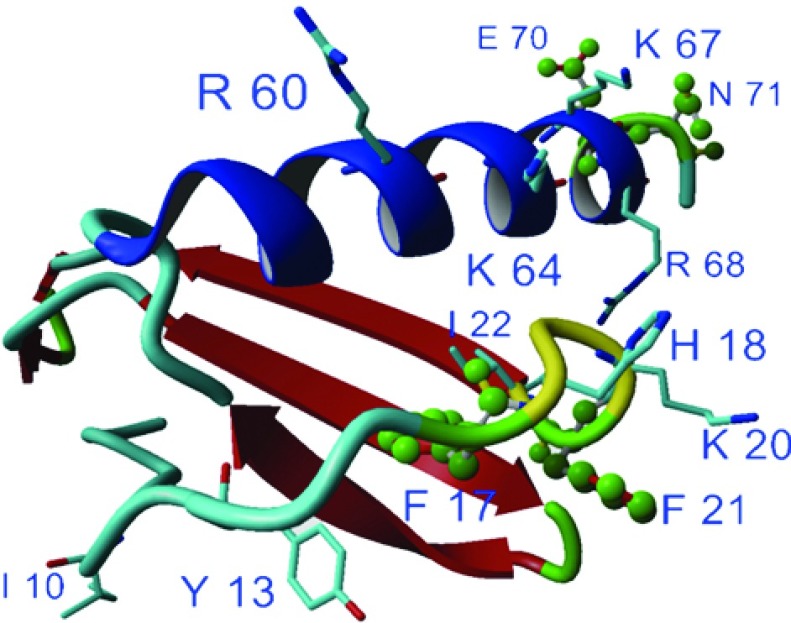
Three-dimensional structure of wtCXCL8 monomer (P10145) using software Yasara for image generation Amino acids involved in receptor binding and GAG binding are highligh-ted. The mutated amino acids of CXCL8(Δ6F17KF21KE70KN71K) are marked in green.

A schematic overview of our structure-based design cycle for dominant-negative chemokine mutants is shown in the flowchart of Figure 1. The *in silico* design process is clearly dependent on the knowledge of the GAG-binding site in the target protein. As our engineering is intended to maintain as good as possible the WT's (wild-type's) GAG ligand specificity, no new GAG-binding sites at different locations of the chemokine have been introduced. We rather aimed to increase the affinity of the existing and well-known GAG-binding site by replacing exposed and structurally independent residues by basic amino acids directly in the GAG-binding site or in its close vicinity (see Figure 2). We have used our molecular model of a CXCL8–heparin complex [13] to prevent interfering with hydrogen bonding and hydrophobic interactions in our mutagenesis protocols in order to keep the chemokine's built-in specificity for a well-defined GAG ligand. However, a slight change in ligand specificity resulted from the mutagenesis procedure cannot be ruled out entirely and will be subject of a subsequent publication (T. Gerlza, B. Hecher and A. J. Kungl, unpublished work). Theoretical GAG-binding affinities are calculated as free energies of binding for the WT protein and the dominant negative mutant. It must be kept in mind, however, that all ligands used for *in silico* docking studies as well as in our *in vitro* binding studies are not the actual GAG ligand of CXCL8 in the human body. Therefore all experiments and calculations presented here must be considered as preliminary. Our engineering approach has already been successfully applied to two chemokines other than CXCL8, namely CCL2 [MCP (monocyte chemoattractant protein)-1] [17] and CCL5 [RANTES (regulated upon activation, normal T-cell expressed and secreted)] [18].

In general, chemokine binding to leucocyte GPCRs is driven mainly by hydrophobic forces. The hydrophobic pocket of CXCL8 is formed by Tyr^13^, Phe^17^, Phe^21^, Ile^22^ and was shown to serve as docking domain for CXCR1 and CXCR2 [14] (see Figure 2). Following initial receptor binding, the N-terminal ELR motif of CXCL8 becomes properly orientated and Glu^4^ and Arg^6^ make electrostatic interactions with the respective GPCR amino acids. The chemokine-binding affinity towards GAGs is primarily driven by electrostatic forces, and it is specificity-guided by hydrogen bonding and van der Waal's interactions. The main GAG-binding site of CXCL8 is located on the C-terminal α-helix (Arg^60^, Lys^64^, Lys^67^ and Arg^68^), forming a cationic cluster, which interacts with the anionic sulphate groups of GAGs. Additional binding involves the chemokine's proximal loop amino acids His^18^ and Lys^20^ [9] (see Figure 2). The most relevant GAG interaction partners for CXCL8 *in vivo* were reported to be HS and CS [19]. Heparin is, however, often used as surrogate GAG *in vitro* and *in vivo* as its overall high degree of sulphation reflects quite well the highly sulphated regions of HS that are the main sites for interactions with proteins such as chemokines. The suggested specific CXCL8-binding domain of HS consists of two sulphated subdomains with a length of six monosaccharides, connected by a chain of 14 low-sulphated monosaccharides [20].

The overlap of the GAG-binding site and the hydrophobic pocket constituting the GPCR interaction site naturally supported our approach to generate non-chemotactic CXCL8 mutants with improved affinity for GAGs. Therefore the first series of our CXCL8 mutants (see Table 1) was designed to contain basic amino acids and combinations thereof at positions Phe^17^ and Phe^21^, in the proximate vicinity of the GAG-binding residues His^18^ and Lys^20^. This was thought to extend the GAG-binding interface and to simultaneously inactivate the GPCR-docking domain, thus preventing the reorientation of the ELR-containing loop needed for receptor activation and triggering. Consequently, three single-site mutants with either lysine at the position 17 or arginine at the position 21 and four double mutants containing different combinations of lysine and arginine at the indicated positions were cloned, expressed and purified. Poly-cations such as poly l-arginine (frequently used as adjuvants in vaccine development) were not considered in our studies because of their unfavourable entropy in ligand recognition and binding studies.

A first structural characterization of these mutants by chaotrope-induced fluorescence shift showed that the proteins were folded in a CXCL8-like manner (results not shown). All mutants exhibited emission maxima between 340 and 350 nm in PBS, which is characteristic for tryptophan fluorescence emission in a solvent-shielded environment typical for folded proteins (the only tryptophan residue of CXCL8, Trp^57^, is located at the top of the C-terminal α-helix pointing into the core of the protein). If denaturing conditions are applied, by adding 6 M guanidine, the previously solvent-protected tryptophan residues become solvent exposed and the emission maximum is shifted significantly to the red. Accordingly, the emission maxima of the denatured mutants were found to be around 360 nm or slightly higher, quite comparable with the emission shift observed for WT CXCL8 thus referring to a similar overall fold.

The mutants’ GAG-binding affinity was determined by IFT for LMWH as well as for unfractionated HS. The *K*_d_ value measured for wtCXCL8 and heparin was found to be 2.28 μM and all mutants with amino acids replacements in the proximal loop (at positions 17 and 21) gave slightly lower affinities (i.e. higher *K*_d_ values) for this GAG ligand (see Table 2). Interestingly, the affinity of wtCXCL8 for HS was 5-fold higher compared with the protein's affinity for heparin, and again all 17/21 mutants gave slightly lower affinities (compared with WT) with the exception of the F21R mutant (Table 2). Since no significant improvement of GAG-binding affinity was achieved with this mutant series, no further investigation of the chemotactic activity of these mutants was carried out. Additional structural characterization of the 17/21 CXCL8 mutants by far-UV CD measurements showed that amino acid replacements in the proximal loop of the chemokine did not affect the secondary structure with respect to the WT (results not shown).

**Table 2:** *K*_d_ of the 1.class CXCL8 mutants, as determined by IFT, compared with wtCXCL8 Ligands were LMWH and unfractionated HS

wtCXCL8 or mutant	*K*_d_ (×10^−6^ M)/LMWH	*K*_d_ (×10^−6^ M)/unfractionated HS
wtCXCL8	2.28±0.51	0.46±0.072
CXCL8(F21R)	5.38±0.54	0.24±0.032
CXCL8(F21K)	2.92±0.27	1.72±0.22
CXCL8(F17K)	3.20±0.23	1.45±0.16
CXCL8(F17RF21R)	4.00±0.53	0.88±0.07
CXCL8(F17KF21R)	4.49±0.41	0.92±0.12
CXCL8(F17RF21K)	2.88±0.29	1.70±0.15
CXCL8(Δ3F17KF21K)	n.d.	n.d.

**Figure 3:**
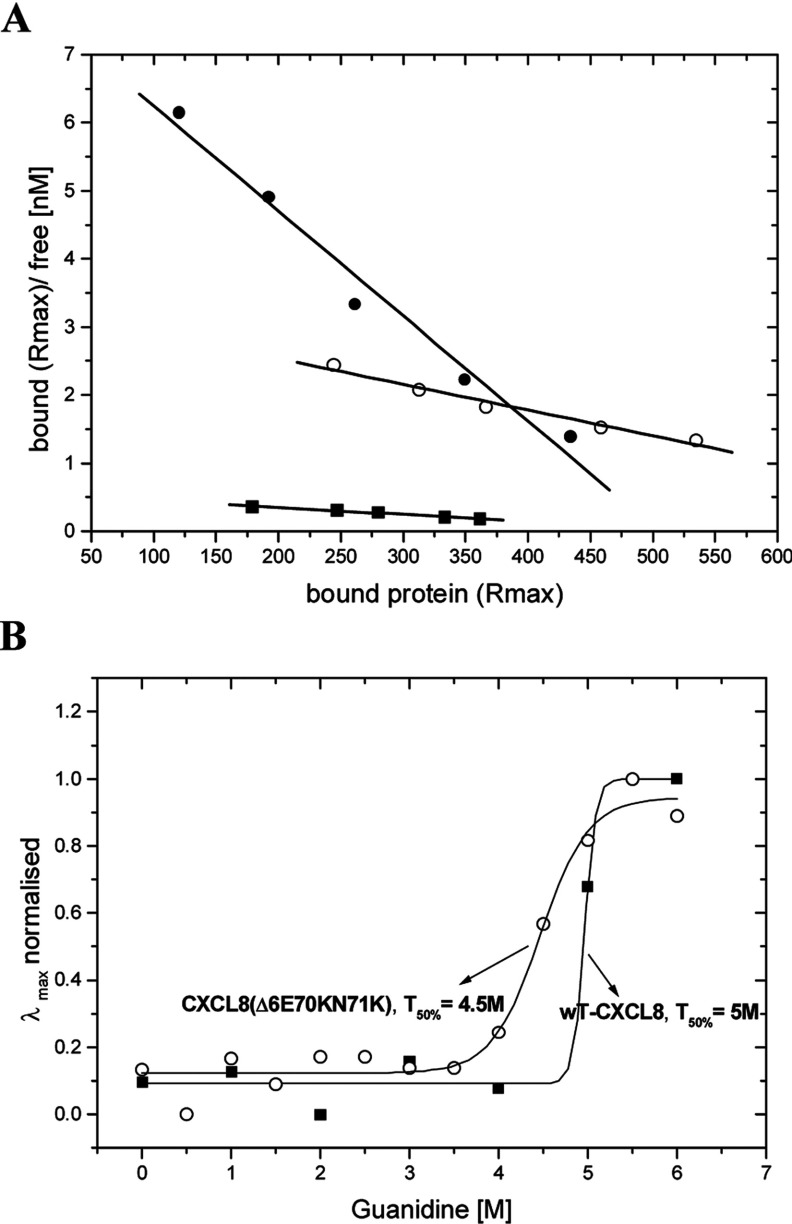
GAG binding and unfolding behaviour of diverse chemokine proteins (**A**) Scatchard plots of HS binding affinities obtained by SPR. wtCXCL8 (■ *K*_d_=1 μM), CXCL8(Δ6E70KN71K) (○ *K*_d_=265nM) and CXCL8(Δ6F17KF21KE70KN71K) (● *K*_d_=65 nM). (**B**) Guanidine-induced unfolding curves of wtCXCL8 and CXCL8(Δ6E70KN71K). Denaturation was monitored by fluorescence wavelength shift and characterized by the transition midpoint of unfolding T_50%_ [*x*_0_ in Eqn. (2)] and by the relative cooperativity [d*x* in Eqn. (2)].

In a second mutagenesis round, we tried to increase the CXCL8–GAG binding affinity by introducing potentially further electrostatic interaction sites into the C-terminal α-helix of the chemokine where the second GAG-binding interface was localized. The second class of our mutants contained therefore an arginine or a lysine residue at position 70 combined with a lysine residue at position 71. Both mutants were designed without the N-terminal ELR motif in order to prevent receptor signalling while keeping the receptor docking domain untouched. For the following biophysical characterization, we concentrated on HS as it is the biologically relevant ligand unlike heparin. With these two new mutants we were able to show a significant impact on GAG-binding affinity. A single mutation in the C-terminal helix [CXCL8(Δ6E70R)], however, was not sufficient to increase the GAG-binding affinity (results not shown). Replacing two amino acids by lysine residues, CXCL8(Δ6E70KN71K), resulted in a >3.5-fold affinity increase for HS binding (see Figure 3A.) The secondary structures of the uncomplexed mutant proteins differed slightly in their helical part. Both double mutants displayed structural changes in the α-helix when incubated with HS, although not as pronounced as wtCXCL8 (results not shown). Overall protein stability was investigated by guanidine-induced unfolding, which showed less cooperativity for mutant unfolding, but with similar transition points of 4.5 M guanidine for the CXCL8(Δ6E70KN71K) mutant compared with 5 M guanidine for the WT (see Figure 3B). The difference in unfolding cooperativity between WT and the CXCL8(Δ6E70KN71K) mutant (dx for the mutant was found to be about 2-fold higher than for the WT; see Figure 3B) is indicative for the coexistence of multiple mutant structural isoforms, each unfolding at slightly different chaotrope concentrations. For CXCL8(Δ6E70R) we were not able to record a fluorescence transition curve since unfolding was not completed at 6 M guanidine. This mutant therefore seems to lack a stable overall protein fold. The chemotactic activity of both double mutants was assessed in a standard Boyden chamber assay using freshly prepared human neutrophils. Both proteins exhibited at concentrations 0.1, 1 and 10 μg/ml the expected knocked-down chemotactic activity compared with wtCXCL8 (results not shown).

The findings obtained from the first two sets of CXCL8 mutants confirmed that we were able to generate chemokine isoforms with higher affinities towards HS only by engineering the C-terminal α-helix. Engineering only the hydrophobic pocket in the proximal loop of the chemokine did not increase the GAG binding. The deletion of the N-terminal six amino acids proved to be sufficient to knock out GPCR activation, as has been shown in previous studies [8].

**Figure 4:**
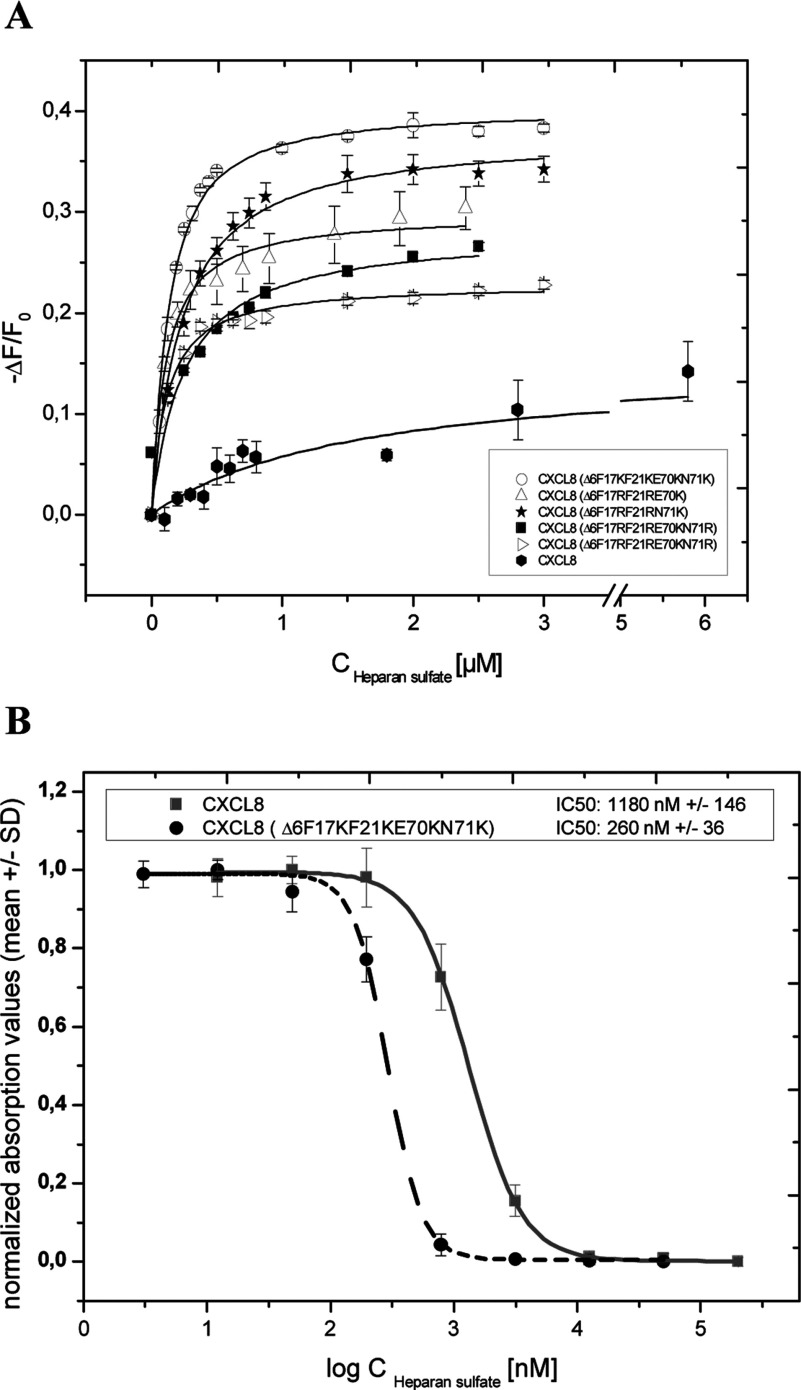
GAG binding and displacement features of the 3.class mutants (**A**) Fluorescence binding isotherms of 3.class CXCL8 mutants compared with wtCXCL8. The changes in fluorescence are plotted as the normalized change in fluorescence (−Δ*F*/*F*_0_, where *F*_0_ is the initial fluorescence intensity before addition of ligand) against the concentration of HS. The data represent mean values of three independent experiments and were fitted as described under Experimental Procedures. (**B**) Displacement of biotinylated wtCXCL8 from HS by CXCL8(Δ6F17KF21KE70KN71K) and unlabelled CXCL8. The normalized absorption values at 450 nM (TMB) are plotted against the concentration of the proteins. The data represent mean values of triplicates measured on one plate and were fitted as described in Experimental Procedures.

Therefore in a last refinement round both GAG-binding sites, the proximal loop and the C-terminal helix, were engineered simultaneously which resulted in a further significant improvement of the GAG-binding affinities for these mutants (all mutants contained, in addition, the Δ6 N-terminal deletion). Five mutants were generated and characterized in the course of this last engineering step. Far-UV spectroscopy showed that three of these mutants exhibited similar secondary structure contents as WT CXCL8, whereas two mutants differed significantly: CXCL8(Δ6F17RF21RN71K) displayed less than 50% of the helical and more than 150% of the CXCL8 sheet content, and CXCL8(Δ6F17RF21RE70KN71R) exhibited more than 200% of the helical but similar sheet content compared with CXCL8. The latter mutant proved, in addition, to be the most stable mutant of this refinement round in guanidine-induced unfolding experiments (see Table 3), indicating that the α-helix is a major structural stabilizing factor in the chemokine. In Table 4 we have summarized the affinity parameters of these five CXCL8 mutants as obtained by fluorescence titration experiments, and in Figure 4(A) we have displayed the respective binding isotherms. As can be seen, the CXCL8(Δ6F17KF21KE70KN71K) mutant showed the best interaction profile with HS with a *K*_d_ value of 97 nM and 38% final fluorescence quenching. Fluorescence quenching observed in the case of the CXCL8 proteins is assumed to be because of direct interaction of the single tryptophan residue with the GAG ligand that leads to fluorescence deactivation. The tryptophan residue of the CXCL8 proteins is located at the C-terminal helix and therefore prone to direct participation in GAG binding. Consequently, a higher final fluorescence quenching value refers to more efficient fluorescence deactivation and thus to tighter binding of the HS ligand. Relating to the proposed displacement mode of action of our CXCL8 mutants, we have performed competition experiments using HS-coated plates for pre-incubation with biotinylated wtCXCL8, which was then displaced by either wtCXCL8 or by the CXCL8(Δ6F17KF21KE70KN71K) mutant (see Figure 4B). In terms of competition efficacy, the mutant was found to have a 4-fold lower IC50 value compared with the WT chemokine.

**Table 3:** Equilibrium denaturation by guanidine

CXCL8 mutant	Transition midpoint (M) guanidine
wtCXCL8	4.96±0.1
CXCL8(Δ6F17RF21RE70K)	2.15±3.6
CXCL8(Δ6F17RF21RN71K)	1.76±0.47
CXCL8(Δ6F17RF21RE70KN71R)	4.51±0.12
CXCL8(Δ6F17RF21RE70RN71K)	3.89±0.26
CXCL8(Δ6F17KF21KE70KN71K)	4.1±0.13

**Table 4:** *K*_d_ and relative saturation of HS-binding sites of the 3.class CXCL8 mutants, as determined by IFTs, compared with wtCXCL8 Ligand: unfractionated HS. A high degree of fluorescence quenching refers to tighter binding of the HS ligand leading to more efficient deactivation of the emission.

wtCXCL8 or mutant	*K*_d_ (×10^−6^ M)/unfractionated HS	Final degree of fluorescence quenching (max. Δ*F*/*F*_0_) in%)
wtCXCL8	0.46±0.072	15
CXCL8(Δ6F17RF21RE70K)	0.103±0.0152	27
CXCL8(Δ6F17RF21RE70RN71K)	0.107±0.0091	22
CXCL8(Δ6F17RF21RE70KN71R)	0.237±0.0614	24
CXCL8(Δ6F17RF21RN71K)	0.209±0.0145	32
CXCL8(Δ6F17KF21KE70KN71K)	0.097±0.0081	38

**Figure 5:**
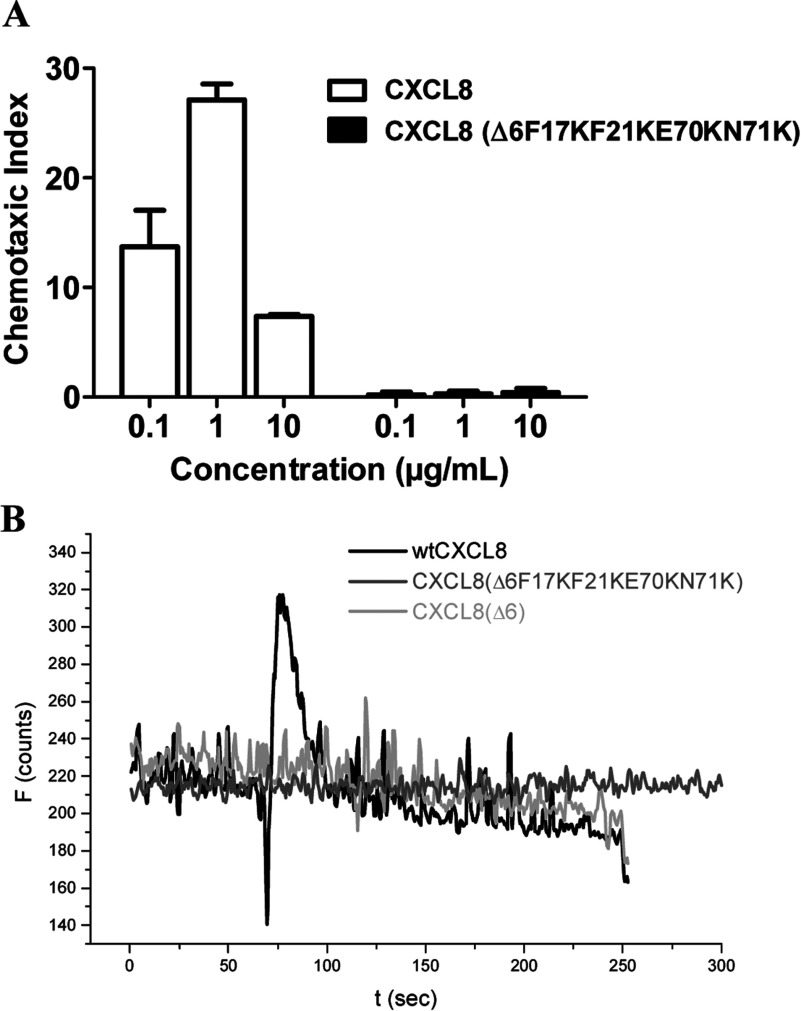
GPCR binding and activation (**A**) Neutrophil chemotaxis at three different wtCXCL8 concentrations compared with CXCL8(Δ6F17KF21KE70KN71K). (**B**) Intracellular Ca^2+^ release as detected by Fura-2AM when freshly prepared human neutrophils were treated with wtCXCL8 (black line),with CXCL8(Δ6F17KF21KE70KN71K) (grey line) or with CXCL8(Δ6) (light grey line).

In Figure 3(A), we have compared the Scatchard plots for HS binding of wtCXCL8, CXCL8(Δ6E70KN71K), and CXCL8(Δ6F17KF21KE70KN71K) as obtained by SPR. The strong increase in the steepness of the interaction curves going from WT via the second mutant generation to the third mutant generation is a clear *in vitro* proof of principle of our engineering method (see Figure 1). With regard to the most probably *in vivo* HS ligand of wtCXCL8, a 5-fold affinity increase was achieved for the CXCL8(Δ6F17KF21KE70KN71K) mutant (using LMWH this increase was even higher, >10-fold, results not shown). In Figure 5(A) we have, in addition, displayed the GPCR knocked-down activity of CXCL8(Δ6F17KF21KE70KN71K). The inverse dose-response profile of neutrophil chemotaxis corresponding to a GPCR down-regulation when using high concentrations of wtCXCL8 is not apparent for CXCL8(Δ6F17KF21KE70KN71K). Both, neutrophil chemotaxis and Ca^2+^ mobilization assays indicate a complete knock-out of CXCR1 and CXCR2 activity of this mutant. In order to verify the missing chemotactic activity to be related to the Δ6 deletion a CXCL8(Δ6) variant was tested in the Ca^2+^ release assay and was found to be inactive (see Figure 5B).

Engineering CXCL8′s proximal loop revealed no significant enhancement for GAG binding, whereas introducing two basic amino acids in the C-terminal helix revealed a 3.5-fold increase in GAG affinity (see Figure 3A). A further enhancement was achieved by combining the two mutation sites with the additional benefit of a complete knock-out of chemotactic activity. 3.class variants containing arginine residues exhibited alterations of secondary structure and/or lack of stability as well as a lower final fluorescence quenching degree (see Table 4). The mutant CXCL8(Δ6F17KF21KE70KN71K), which was shown to have the highest GAG-binding affinity in combination with the highest degree of final fluorescence quenching and totally knocked-out chemotactic activity, emerged as the potentially most potent dominant-negative CXCL8 mutant from the *in vitro* characterization experiments and was therefore further investigated for its *in vivo* activity in murine acute inflammatory models.

CXCL8 is a key chemokine up-regulated in the serum of RA (rheumatoid arthritis) patients and pathology-specific glycans providing high affinity-binding sites for CXCL8 on the inflamed endothelium have been identified in RA patients [21]. Moreover, neutrophils are the most abundant leucocyte population present in the joint of RA patients with active RA [22]. The mouse equivalents of CXCL8 responsible for neutrophil recruitment are CXCL1 [KC (keratinocyte chemoattractant)] and CXCL2 [MIP (macrophage inflammatory protein)-2]. In a first set of *in vivo* experiments the efficacy of CXCL8(Δ6F17KF21KE70KN71K) was tested in a mouse model of acute inflammation, induced by murine CXCL1 (KC). Intra-articular injection of CXCL1 resulted in a significant cell recruitment (mostly neutrophil) into the knee cavity which could be dose-dependently inhibited by subcutaneous administration of a CXCL8(Δ6F17KF21KE70KN71K) (results not shown). This initial experiment was performed to verify that a human CXCL8 decoy protein was able to act as anti-inflammatory agent in an inflammatory model induced by the mouse functional homologue of CXCL8. Our data are in line with a previous publication that showed that human CXCL8 was able to induce sustained inflammatory response in a murine air-pouch model [24], indicating that mice are a suitable species to test therapeutic agents that interfere with the CXCL8 axis.

**Figure 6:**
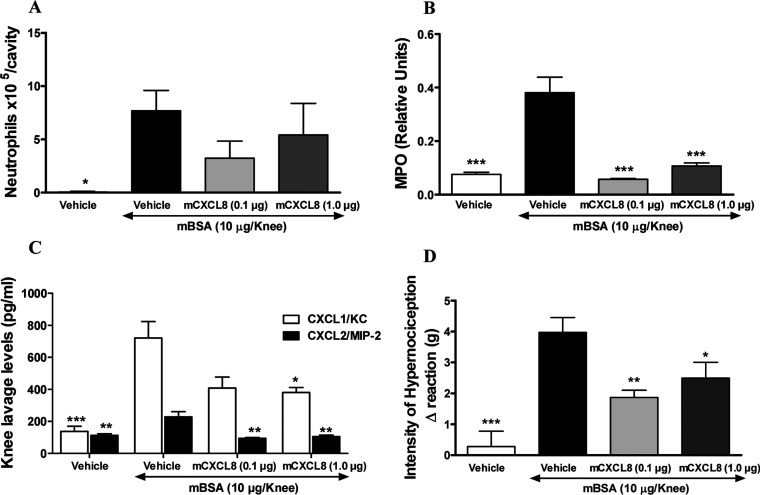
Investigating CXCL8(Δ6F17KF21KE70KN71K) in the mBSA-induced arthritis model CXCL8(Δ6F17KF21KE70KN71K) induced partial inhibition of neutrophil migration into the murine joint (**A**) and significant inhibition into surrounding tissue as assessed by MPO activity measurement (**B**) as well as CXCL1/KC and CXCL2/MIP-2 levels in knee lavage (**C**) in a model of antigen (mBSA)-induced arthritis. (Note: CXCL8(Δ6F17KF21KE70KN71K) is referred to as mCXCL8 in all panels in Figure 6.) In this model a significant reduction in degree of hypernociception was also observed (**D**) *P<0.05; **P<0.01 and ***P<0.001 against mBSA vehicle treated group.

**Figure 7:**
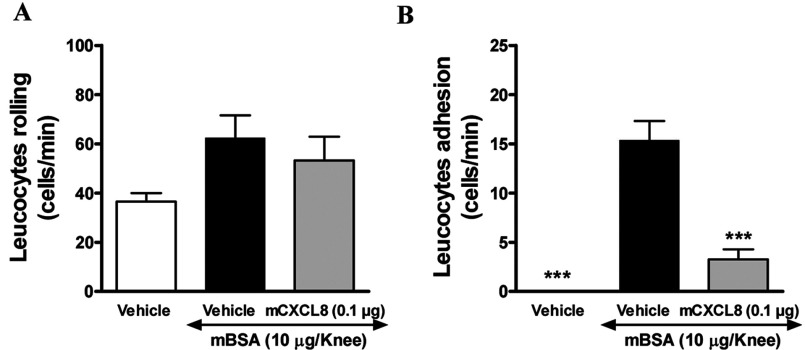
Effects of CXCL8(Δ6F17KF21KE70KN71K) on leucocyte rolling and adhesion Effect of CXCL8(Δ6F17KF21KE70KN71K) on leucocyte rolling (**A**) and adhesion (**B**) in a model of antigen (mBSA)- induced arthritis assessed by intravital microscopy (Note: CXCL8(Δ6F17KF21KE70KN71K) is referred to as mCXCL8.) ***P<0.001 against mBSA vehicle treated group.

We have subsequently evaluated CXCL8(Δ6F17KF21KE70KN71K) efficacy in a more complex model of AIA (antigen induced arthritis). CXCL8(Δ6F17KF21KE70KN71K) subcutaneous administration at the doses of 0.1 and 1.0 μg/mouse was able to reduce neutrophils recruitment to the knee cavity (Figure 6A), however, without reaching statistical significance due to the high inter-individual variability. This was probably due to the difficulty to properly collect knee lavage in this model because of the severity of inflammatory response that involved also the surrounding tissue. A significant reduction in neutrophil infiltration into the peri-articular tissues, as evaluated by MPO assessment, was however observed at both doses (Figure 6B), suggesting that a plateau effect is reached at the 0.1 μg dose. Moreover, CXCL8(Δ6F17KF21KE70KN71K) treatment reduced murine CXCL1 (KC) and CXCL2 (MIP-2) levels in the knee tissue (Figure 6C). In addition to the influx of neutrophils into the joints, AIA is known to induce a significant hypernociception in mice. CXCL8(Δ6F17KF21KE70KN71K) was able to significantly diminish also the extent of inflammation-related hypernociception (Figure 6D). Finally, in order to evaluate whether the observed effects of CXCL8(Δ6F17KF21KE70KN71K) were because of its ability to prevent interaction of leucocyte with the synovial microvessels, an intravital microscopy experiment was conducted. CXCL8(Δ6F17KF21KE70KN71K) treatment was performed at the dose of 0.1 μg/mouse subcutaneously, since no obvious increased effects were observed with the 1.0 dose in any of the measured parameter in the previous experiment. CXCL8(Δ6F17KF21KE70KN71K) had no effect on leucocyte rolling, while dramatically reduced their adhesion (Figures 7A and 7B). The data obtained in these studies clearly show that subcutaneous treatment with CXCL8(Δ6F17KF21KE70KN71K) significantly impacts on two major aspects of arthritis: neutrophil recruitment and inflammation-related hypernociception, reducing the levels of two chemokines relevant for neutrophil recruitment, murine CXCL1 (KC) and CXCL2 (MIP-2). The intravital microscopy data further indicate that the compound acts via inhibition of leucocyte adhesion on the venule at the site of joint inflammation, resulting in inhibited leucocyte transmigration into the knee cavity and surrounding tissues. These data support our proposed mechanism of action for CXCL8(Δ6F17KF21KE70KN71K) as a CXCL8-based decoy protein antagonising the interaction of the WT chemokine with its GAG coreceptor.

The immunogenic potential of CXCL8(Δ6F17KF21KE70KN71K) compared with wtCXCL8, as well as CXCL8(Δ6F17KF21KE70KN71K) and wtCXCL8 derived peptides, was assessed using an *in vitro* T-cell activation assay. In conclusion, slightly higher immunogenic responses were observed for CXCL8(Δ6F17KF21KE70KN71K) and CXCL8(Δ6F17KF21KE70KN71K) derived peptides in the *in vitro* T-cell activation assay, as compared with wt CXCL8. At the population level; however, these differences were not statistically significant.

## CONCLUSION

GAGs are an abundant class of O-linked, highly sulphated polysaccharides, which drive and control protein activity by interacting with basic amino acids on the target protein. Interfering with these functional interactions seems to be a potential way of antagonising the target protein's pathological role. We have recently reviewed the various molecular approaches of interfering with GAG-protein interactions [23]. We have chosen the protein decoy path because, if a certain degree of specificity can be assumed between a chemokine and its GAG ligand [25], CXCL8 should have a built-in specificity for its GAG ligand. Since the exact structure of the CXCL-8-specific GAG ligand is unknown, although there have been suggestions for such a structure [20], it seemed therefore to be a straightforward way to build on the chemokine's ability to recognize its natural GAG ligand and try to improve its affinity. If maintenance of hydrogen bonds and hydrophobic interactions can be achieved, both of which drive the specificity between proteins and GAGs, our engineering approach seemed to be a novel and effective way to generate potent and selective GAG antagonists – on a protein basis. In the present paper, we have applied this engineering process to CXCL8 and after three mutation rounds we ended up with a structurally stable, dominant negative protein, CXCL8(Δ6F17KF21KE70KN71K), which exhibited a 5-fold improvement in GAG-binding affinity and which inhibited neutrophil recruitment into inflamed joints as well as hypernociception *in vivo*.

The active dose of CXCL8(Δ6F17KF21KE70KN71K) in this AIA model was found to be very low (0.1 μg/mouse, corresponding to about 4 μg/kg) referring to a very efficient displacement mode by the decoy human chemokine, which might translate into low effective doses in human clinical trials.
